# Pre-hospital emergency: analyzing the status of medication and equipment in pre-hospital emergency bases

**Published:** 2025-01

**Authors:** Sajad Noorian, Najmeh Chegini, Mohadese Aliakbari, Mostafa Amiri, Mojtaba Senmar

**Affiliations:** ^*a*^ Department of Statistics, Faculty of Science, University of Qom, Qom, Iran.; ^*b*^ Social Determinants of Health Research Center, Research Institute for Prevention of Non-Communicable Diseases, Qazvin University of Medical Sciences, Qazvin, Iran.; ^*c*^ Emergency Medical Service Center, Qazvin University of Medical Sciences, Qazvin, Iran.

**Keywords:** Emergency Medical Services, Analysis, Equipment Failure, Medication Systems

## Abstract

**Background:**

Pre-hospital emergency care is a comprehensive system that responds to the medical needs of injured patients outside of health care centers. The right equipment and facilities are necessary to provide proper and satisfactory services. Due to the increase in pre-hospital emergency services and the high prevalence of emergency events, the present study was conducted to investigate the status of medication and equipment in pre-hospital emergency bases and compare it with national standards.

**Methods:**

This descriptive-analytical study was conducted in Iran in 2022-2023. Sampling was done in 46 urban and road bases using the census method. Data collection was done using two checklists. The first checklist, in 17 sections, was based on the latest edition of the instructions for medication and equipment of Iran's pre-hospital emergency organization. The second checklist included the speci-fications of the base. The collected data were analyzed using descriptive and analytical statistics and SPSS25 software.

**Results:**

Of the 46 bases included in the study, 41.3% were urban bases and the rest were road bases. The mean age for building the bases was 13.76±9.47 years (the minimum was one year and the maximum was 45 years). The mean score of the status of medication and equipment in the bases was 224.34 ± 32.05 (minimum score 155 and maximum 322), which was 56.09% of the total score. The highest score was obtained in the medication section of the patient cabin (81.88%) and the lowest score was in the CBRNe bag equipment section (4.55%).

**Conclusions:**

The results showed that the status of the medication and equipment of the pre-hospital emergency bases is far from the standards. This long distance can affect the quality of pre-hospital emergency services. Resolving deficiencies and reaching standards requires more attention.

## Introduction

Humans are exposed to injuries caused by trauma, cardiovascular diseases, and various events.^1^ Every year, approximately 5 million people in the world die due to injuries caused by traffic accidents, violence, burns, falls, etc.^2^ In addition, millions of people suffer from a disability. The noteworthy point is that the amount of damage caused by accidents is much higher in developing countries.^2^ Therefore, saving the lives of trauma victims and pa-tients suffering from internal diseases such as strokes, convulsions and decreased levels of consciousness is al-ways a major concern of the people and the health system.^1^ For this reason, it can be said that pre-hospital emer-gency is of considerable importance as the first line of care and treatment system in dealing with patients.^3^

The pre-hospital emergency is a comprehensive care system that responds to the medical needs of injured patients outside of medical care centers.^3^ In Iran's pre-hospital emergency, known as 115, services are free.^4^ Although only three decades have passed since the establishment of this system in Iran, there is much evidence that it has provided valuable services to the health system in this short time.^5^ Currently, the growth rate of pre-hospital emergency missions is 16%, and in other words, the number of missions of this system doubles almost every 6 years.^4^ Therefore, it should be said that this system plays an essential role in preserving human life.^6^ One of the goals of this system is to provide the right treatment at the right time and place by using the available resources.^5^ But the success of this system depends on the skill level of personnel, the ability of responsible people, trained staff, and facilities.^6^ Apart from this, pre-hospital emergency centers need appropriate equipment and facilities to provide proper and satisfactory services.^1^

Specialized and new equipment and facilities are needed in pre-hospital emergency centers to provide appropriate services because the traditional emergency parameters have changed with the advancement of medical technology.^7^ In emergency care, specialized equipment can make a difference in whether the patient receives medical treatment in the golden time or not.^8^ Therefore, the importance of medical equipment and advanced technologies in follow-up, diagnosis, treatment, and research in healthcare is not hidden from anyone.^8^ Patient care equipment, which has a variety of simple and small to complex and large,^9^ is an important factor in the effectiveness and efficiency of services.^2^ These are very important assets that significantly contribute to improving the quality of healthcare services.^10^

Currently, due to various factors, the use of pre-hospital emergency services is increasing day by day.^11^ With this in mind, the failure of medical equipment may affect the effectiveness of healthcare services, causing severe harm to patients and the environment.^10^ Apart from this, lack of equipment is one of the challenges of pre-hospital emergency.^2^ Therefore, assessing the condition of medical equipment is an important activity during the maintenance and management of the lifecycle of the equipment to increase availability, performance, and safety.^10^ Another component of pre-hospital care is medication administration.^12^ It should be said that prescribing medicine for injured and needy patients is one of the duties of intermediate and advanced emergency technicians who treat many patients and injured every year whether they like it or not.^13^ It is quite obvious that people who use medicine to treat patients, in addition to having accurate and correct information, must also have reliable medical resources to be able to take the first step in treatment correctly and confidently.^13^ Although the standards of medications and equipment have been provided to pre-hospital emergency centers in Iran,^8,14^ the medications and equipment are not in good condition.^15,16^ Studies in Iran have also reported that the condition of medical and non-medical equipment is far from the standards and is not in a favorable condition.^14,16^ Another study in Uganda also showed that the average condition of the equipment in pre-hospital emergency is lower than the standard.^17^ Although the standards are different in different countries, studies in Japan and Norway also showed that some medications and equipment in the pre-hospital emergency do not have good condition.^18,19^

Although medical equipment is the most important and essential tool in the process of the health and treatment system, and a major share of the costs of this system is allocated annually for the purchase, maintenance, and repair of equipment,^12^ few studies have investigated the status of pre-hospital emergency equipment paid. Therefore, due to the importance of the subject and the lack of sufficient studies in this field, the present study was conducted to analyze the Existing Status of Medication and Equipment in Pre-Hospital Emergency Bases.

## Methods 

**Setting and study design**

The descriptive-analytical study was conducted 2022-2023 in Qazvin province, Iran. According to the latest national census, Qazvin province has a population of 1,273,761 people, of which 952,149 people live in urban areas and the rest live in rural areas. All the pre-hospital emergency bases of Qazvin province (46 bases) formed the research environment. Sampling was done by census method. All the checklists were completed by the researchers and by referring to the provincial databases. Checklists were completed in morning and evening shifts. The criterion for inclusion in the study was the operational activity of the base at the time of the research.

**Data Collection Tools**

Data was collected using two checklists: the first part of the checklist includes questions about the name of the city in which the base is located (including Qazvin, Takestan, Abik, Buin Zahra, Avaj, and Alborz cities), the type of base (urban, road), the name of the base (46 bases), type of base structure (conex, building) and base establishment date (year). 

The second part of the checklist was based on the latest edition of the instructions for medication and equipment of Iran's pre-hospital emergency organization (including 400 items).^20^

In this checklist, all the medication and equipment needed inside the ambulance and the pre-hospital emergency base are listed in 17 sections. The items include patient cabin equipment (41 items), patient cabin medications (3 items), jam-bag equipment (58 items), jam-bag medications (42 items), reserve bag equipment (15 items), reserve bag medications (5 items), equipment Delivery bag (16 items), delivery bag medications (3 items), triage bag equipment (11 items), dressing depot bag equipment (13 items), chemical, biological, radiological, nuclear and explosive (CBRNe) bag equipment (22 items), personal protective equipment (12 items), cabin equipment (74 items), base medications (49 items), safety and technical service equipment (18 items), traffic detection and management equipment (4 items) and front cabin equipment (14 items). In the observation of equipment and medication, number one was considered for the presence or health, and zero for the absence and incompleteness.^8,14^ The questions and subsets of this checklist are exactly the latest version of the standards of the country's emergency organization. The aforementioned checklist has been used in various studies in Iran and the validity and reliability of the tool have been checked and confirmed in the Iranian society.^8,16^ In the present study, the reliability of the tool was obtained using Cronbach's alpha of 0.85.

**Statistical methods**

After collecting the data and entering them into the software, the statistical analysis of the data was done using SPSS version 25 software and using descriptive and analytical statistics.

## Results

Of the 46 bases included in the study, 41.3% were urban and the rest were road bases. 91.3% of the bases have building structures and the rest are in the form of concourses. 58.8% of the bases were owned by the university. The mean age of building bases was 13.76 ± 9.47 years (the minimum was 16 years and the maximum was 45 years). The overall mean score in the section on the status of medication and equipment in the bases was 224.34 ± 32.05 (minimum score 155 and maximum 322). The mean score of the medications section of the patient cabin was (2.45 ± 0.58, minimum 1 and maximum 3), which received 81.88% and was the highest score among 17 sections (Table 1). The mean score of equipment condition was 0.56 ± 0.06in urban bases and 0.55 ± 0.09 in road bases (Table 2). The mean score of the equipment in the bases that had building structures was 0.56 ± 0.08 and in the conex box bases was 0.53±0.06. The results showed that the mean score of patient cabin equipment, patient cabin medications, reserve bag medications, delivery bag equipment, triage bag equipment, personal protection equipment, base medications, safety and technical service equipment in Takestan City was significantly different from other cities (p=0.019, p=0.017, p=0.004, p=0.005, p=0.016, p=0.021, p=0.012, p=0.044).

**Table 1:** Mean and standard deviation of medication and equipment.

Section	Mean	Std. Deviation	Minimum	Maximum	Percent	Total number of items
Patient Cabin Equipment	23.48	2.66	14.00	28.00	57.26%	41
Patient Cabin Medications	2.46	0.59	1.00	3.00	81.88%	3
Jambag Equipment	43.80	3.19	36.00	51.00	75.52%	58
Jambag Medications	29.43	2.53	23.00	34.00	70.08%	42
Reserve Bag Equipment	5.74	6.77	0.00	15.00	38.26%	15
Reserve Bag Medications	1.89	2.40	0.00	5.00	37.83%	5
Equipment Delivery Bag	11.76	3.87	3.00	16.00	73.51%	16
Delivery Bag Medications	1.39	1.08	0.00	3.00	46.38%	3
Triage Bag Equipment	3.09	2.43	0.00	8.00	28.06%	11
Dressing Depot Bag Equipment	3.50	4.66	0.00	11.00	26.92%	13
CBRNe Bag Equipment	1.00	2.56	0.00	14.00	4.55%	22
Personal Protective Equipment	5.46	2.12	0.00	9.00	45.47%	12
Cabin Equipment	42.00	18.49	0.00	72.00	56.76%	74
Base Medications	30.89	4.30	23.00	39.00	63.04%	49
Safety and Technical Service Equipment	7.87	2.66	0.00	12.00	43.72%	18
Traffic Detection and Management Equipment	0.89	0.67	0.00	3.00	22.28%	4
Front Cabin Equipment	9.70	1.33	5.00	12.00	69.25%	14
Total	224.35	32.05	155.00	312.00	56.09%	400

**Table 2:** Mean and standard deviation of the general status of medication and equipment based on the type of base.

	Group Statistics			Std. Deviation	Std. Error Mean
	type of base	N	Mean		
Patient Cabin Equipment	Urban	19	.57	.03	.008
	Road	27	.57	.07	.01
Patient Cabin Equipment	Urban	19	.77	.22	.051
	Road	27	.85	.16	.032
Jambag Equipment	Urban	19	.75	.05	.012
	Road	27	.75	.05	.010
Jambag Equipment	Urban	19	.69	.06	.014
	Road	27	.70	.05	.011
Reserve Bag Equipment	Urban	19	.42	.46	.10
	Road	27	.35	.44	.08
Reserve Bag Equipment	Urban	19	.41	.49	.11
	Road	27	.35	.47	.09
Equipment Delivery Bag	Urban	19	.73	.21	.05
	Road	27	.73	.26	.05
Equipment Delivery Bag	Urban	19	.35	.35	.08
	Road	27	.54	.34	.06
Triage Bag Equipment	Urban	19	.18	.23	.05
	Road	27	.34	.18	.03
Triage Bag Equipment	Urban	19	.22	.34	.07
	Road	27	.29	.37	.07
CBRNe Bag Equipment	Urban	19	.03	.08	.01
	Road	27	.05	.13	.02
Personal Protective Equipment	Urban	19	.43	.16	.03
	Road	27	.46	.18	.03
Cabin Equipment	Urban	19	.65	.16	.03
	Road	27	.50	.28	.05
Base Medications	Urban	19	.61	.09	.02
	Road	27	.64	.08	.01
Safety and Technical Service Equipment	Urban	19	.38	.16	.03
	Road	27	.47	.12	.02
Traffic Detection and Management Equipment	Urban	19	.17	.14	.03
	Road	27	.25	.17	.03
Front Cabin Equipment	Urban	19	.67	.11	.02
	Road	27	.70	.07	.01
Total Mean Score	Urban	19	.56	.06	.01
	Road	27	.55	.09	.01

## Discussion

The present study, which analyzed the status of pre-hospital emergency medication and equipment in Qazvin province, showed that the status of pre-hospital emergency medication and equipment is far from the standards. So only 56.09% of marks have been obtained. The highest score was in the medication and equipment section of the base, jam-bag equipment, and delivery bag equipment. This means that there is the least deficiency and the lowest score was in the CBRNe equipment section. In the sense that we saw the greatest lack of equipment in this domain. In addition, the condition of the equipment in the urban bases was better than that on the road. Bases that had building structures scored higher than concourse structures. According to the researcher, who has years of experience working in this system, one of the reasons for the difference between urban and road bases is the proximity of urban bases to pre-hospital emergency decision-making centers. Although it is not far-fetched that the situation is better in urban bases that face high population density, this difference can have an impact on how to provide services and increase the quality of care in road areas. Regarding the structure, it should be noted that when a structure is in the form of a building, it naturally has more space and environment to provide facilities and equipment. Apart from this, it is easier to maintain medical and non-medical equipment in these types of structures than bases with conex box structures. Although these reasons have been stated from the researcher's point of view and experiences in this system, it should be noted that the status of medications and equipment in this system should not be different from each other at different points. In some studies, lack of equipment and facilities has been raised as one of the most important challenges of pre-hospital emergency.^21,22^ In both of the above qualitative studies, the participants mentioned the lack of equipment and facilities, which is somehow consistent with the findings of this study. Although the above studies have been done qualitatively, for accurate judgment and statistical analysis, this part should have been done quantitatively and using numbers. The scores obtained in the medication and equipment section of the current study confirm this deficiency.

The study of Moradi et al.^1^ in Khorramabad, Iran showed that the mean score obtained in this province (48%) is lower than the mean score obtained in the present study. Although the scores obtained in Moradi's study are low, it must be said that this difference is unsigficanted, and somehow in both studies the general condition of medication and equipment is not suitable and is far from the standards. Considering the location of the two studies, it can be said that one of the reasons for the unfavorable condition of medications and equipment in these two provinces is economic issues and limitations. In interpreting the results, it should be considered that development is influenced by financial possibilities. Considering that the pre-hospital emergency department does not have a revenue-generating nature and is a service sector, the development and improvement of the conditions in this system can be affected by the financial and economic restrictions of the Ministry of Health. The study of Kazem Nejad et al.^14^ showed that the mean score of equipment in pre-hospital emergency ambulances is 66.5%, which is higher than the scores of the present study. It seems that the condition of the ambulances in this study was better than in the present study. The important point is that Pourshikhian et al. only considered the bases of one city with 13 ambulances, and in the current study, the entire province with 46 bases was included. It seems that the increase in the sample size has brought the results closer to reality. In addition, it should also be taken into consideration that Rasht is one of the busiest provinces in Iran in terms of its geographical location, which accommodates a large number of travelers every year.

Ningwa et al.'s study in Uganda^17^ showed that ambulances and emergency units in health centers lacked the most basic equipment and medicines to monitor and treat emergency conditions. This weakness in medicine and equipment has been observed at all levels of the health system, regardless of the level or ownership of the center or ambulance. Although the above study has used different standards and criteria, it seems that in both studies we are facing shortages and deficiencies in facilities and equipment. The important point in interpreting the findings of the above study and the present study is that it seems that the less economic development of both countries can be one of the reasons for the limitations in facilities and equipment. Although Rad et al.^8^ have used different standards to check the condition of the equipment, the results of the two studies differ significantly; Because in Rad et al.'s study, 71.68% of the equipment is present in all bases. The significant point in the interpretation of the data is that the above study only studied 5 urban bases; While in the present study, all urban and road bases of the province have been investigated. Apart from this, different standards can also explain the difference in the results of the two studies. In a study conducted in Nepal,^23^ the results showed that more than half of the ambulances had less than the mean equipment for basic life support. Although the above study only considered the basic life support equipment; It seems that the results of both studies confirm the lack and weakness of having these pieces of equipment. The important point is that the standards of the two studies are different because the standards of the Ministry of Health of Iran were used in the researchers' study. It seems that the economic situation and problems related to the development of the countries in the present study and the above studies in low-income and developing countries are the same, so this has led to the similarity of the results of the studies.

The results in the CBRNe bag, triage bag, and personal protection equipment section are also significant. A CBRNe incident is an emergency that can result in injury, illness, or loss of life. The emergency unit as a health system is at the forefront of CBRNE response and the employees act as the first responders,^17^ but in the present study, the condition of the CBRNe bag equipment has been assigned the lowest percentage; And this serious shortage can affect the safety and security of personnel in the event of such events. In line with the findings of the present study, Moradi et al.'s study^1^ also obtained the lowest score for the CBRNe bag equipment. It seems that due to the fact that biological, chemical, nuclear, and radiological accidents happen less often and the personnel and management system has faced these accidents less, it has attracted less attention. This interpretation could be one of the reasons for the weakness in this category. Another important issue is that although there are many recommendations related to use personal protective equipment to prevent diseases and maintain the safety of pre-hospital emergency technicians, in the current study, personal protective equipment has a mean of 45.47%. Contrary to the present study, Sabouri et al.'s study^24^ showed that the greatest deficiency is in the field of personal protective equipment (17.94 %); Although both studies show a great gap in the standards in this field. One of the main and important tasks in prehospital emergency care is the accurate triage of patients. prehospital emergency triage may play a more crucial role, especially in low- and middle-income countries. However, the current study showed that there is a large gap in the standards in the field of triage equipment and the condition of the equipment shows a serious deficiency, which is in line with the findings of some studies.^1,25^

The organization and provision of pre-hospital emergency services vary from country to country and sometimes between regions within the country.^26^ The important and noteworthy point is that in a system that has a vital role in improving outcomes in medical and obstetric emergencies, severe injuries, and other time-sensitive diseases,^17^ Pre-hospital emergency bases need proper equipment and facilities to provide satisfactory services, but the literature review showed that the challenge of lack of facilities, medication, and equipment is more important in developing countries. While the presence of well-equipped and ready ambulances can play the biggest role in saving the patient's life and reducing casualties.^1^ It seems that pre-hospital emergency is less developed in low-income countries.^27^ According to the researcher, apart from paying attention to economic issues and existing limitations in supplying equipment and medications, the management and organization system plays a very important role in pre-hospital emergencies. Having a proper relationship with headquarters managers, using management principles in the arrangement of operational forces, and building and opening bases according to needs assessment and reasonable criteria can bring the status of drugs and equipment in this structure closer to the standards.

## Conclusion

Although medication and equipment in the pre-hospital emergency have a fundamental and undeniable role in providing correct, accurate, and timely services, the present study showed that the condition of the medication and equipment in the pre-hospital emergency of Qazvin province is far from the national standards. It is clear that a lack of medicine and equipment not only reduces the quality of care and the effectiveness of service delivery but also can lead to harm to the recipients of these services. Since this system is related to seconds, any deficiency can have adverse consequences for society and people's lives. Managers and policymakers are expected to periodically evaluate medication and equipment with proper planning based on national standards and take steps to improve the conditions by providing the necessary facilities and equipment. Considering the importance of the role of pre-hospital emergency, more investment should be made to improve the status of medications and equipment in this system, and apart from the use of national standards, international standards should also be considered. The shortage and defects in medicine and equipment in this system disrupt the proper care process and increase the cost of treatment and care. It is recommended to conduct more studies to investigate the relationship between the status of medication and equipment and the outcomes of patient care and treatment.

**Acknowledgement**

We would like to thank the Research Vice-Chancellor of Qazvin University of Medical Sciences and the people who helped in this study.

**Authors' contributions**

M.S. and M.A. designed the study. N.C. and M.A. participated in data collection. S.N. and M.S. analyzed and interpreted the data. All author wrote the main manuscript text. Final approval of manuscript by all authors.
